# Privacy Concerns Related to Data Sharing for European Diabetes Devices

**DOI:** 10.1177/19322968231210548

**Published:** 2023-11-13

**Authors:** Pietro Randine, Matthias Pocs, John Graham Cooper, Dimitrios Tsolovos, Miroslav Muzny, Rouven Besters, Eirik Årsand

**Affiliations:** 1 Department of Computer Science, Faculty of Science and Technology, UiT The Arctic University of Norway, Tromsø, Norway; 2 Norwegian Centre for E-health Research, University Hospital of North Norway, Tromsø, Norway; 3 Stelar Security Technology Law Research, Hamburg, Germany; 4 Norwegian Quality Improvement of Laboratory Examinations, Haraldsplass Deaconess Hospital, Bergen, Norway

**Keywords:** security, privacy, software as medical device, GDPR, medical device

## Abstract

**Background:**

Individuals with diabetes rely on medical equipment (eg, continuous glucose monitoring (CGM), hybrid closed-loop systems) and mobile applications to manage their condition, providing valuable data to health care providers. Data sharing from this equipment is regulated via Terms of Service (ToS) and Privacy Policy documents. The introduction of the Medical Devices Regulation (MDR) and In Vitro Diagnostic Medical Devices Regulation (IVDR) in the European Union has established updated rules for medical devices, including software.

**Objective:**

This study examines how data sharing is regulated by the ToS and Privacy Policy documents of approved diabetes medical equipment and associated software. It focuses on the equipment approved by the Norwegian Regional Health Authorities.

**Methods:**

A document analysis was conducted on the ToS and Privacy Policy documents of diabetes medical equipment and software applications approved in Norway.

**Results:**

The analysis identified 11 medical equipment and 12 software applications used for diabetes data transfer and analysis in Norway. Only 3 medical equipment (OmniPod Dash, Accu-Chek Insight, and Accu-Chek Solo) were registered in the European Database on Medical Devices (EUDAMED) database, whereas none of their respective software applications were registered. Compliance with General Data Protection Regulation (GDPR) security requirements varied, with some software relying on adequacy decisions (8/12), whereas others did not (4/12).

**Conclusions:**

The study highlights the dominance of non-European Economic Area (EEA) companies in medical device technology development. It also identifies the lack of registration for medical equipment and software in the EUDAMED database, which is currently not mandatory. These findings underscore the need for further attention to ensure regulatory compliance and improve data-sharing practices in the context of diabetes management.

## Introduction

People with type 1 and type 2 diabetes mellitus often have a wide range of devices and digital health applications (apps) available to help them manage their diabetes.^1^ These can support lifestyle and pharmacological interventions, eg, devices such as blood glucose meters, continuous glucose monitoring (CGM) devices, insulin pumps, hybrid closed-loop systems, smart insulin pens, and associated apps.^2,3^

In Europe, medical equipment for chronic diseases like diabetes may be distributed to patients based on national agreements between health authorities and device producers. These agreements are valid for all citizens covered by national health insurance in most European Economic Area (EEA) countries. Data from diabetes devices and apps can provide crucial input to health care providers (HCPs) when they assess risk factors, review treatment plans, and assess patient well-being at periodic medical assessments.^4-6^

### What is a Medical Device in Europe?

The definition of a medical device in the European market is outlined in the Medical Device Regulation (MDR), which became effective on May 26, 2021.^7^ The MDR’s definition of “device” includes standalone software that meets certain criteria, such as being designed to diagnose, prevent, monitor, predict, prognosis, treat, or alleviate disease. Another regulation related to medical devices is the In Vitro Diagnostic Regulation (IVDR), established in 2017,^8^ which governs medical devices related explicitly to tests performed outside of a living organism.

European Commission, in conjunction with the new regulations (MDR and IVDR), has also established a database called the European Database on Medical Devices (EUDAMED), aiming to enhance traceability, cooperation, and transparency within the medical device sector.^9^ Participation in this database is currently voluntary and will become mandatory in all its components in 2026.^10^

### General Data Protection Regulation and Other Standards

The General Data Protection Regulation (GDPR) is the most prominent European regulation, established in 2016, that concerns data protection and privacy in EEA.^11^ In addition to GDPR, individual countries may have their own national regulations for sensitive data, which are particularly relevant for the medical domain (GDPR—Article 9).

**Figure 1.**
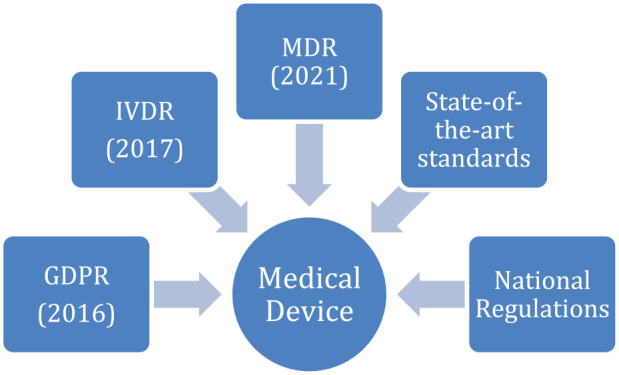
Regulations and standards affecting medical devices in EEA. Abbreviations: EEA, European Economic Area; GDPR, General Data Protection Regulation; IVDR, In Vitro Diagnostic Medical Devices Regulation; MDR, Medical Devices Regulation.

Thus, the global picture is exceptionally complex, with various international standards concerning technological aspects (see Figure 1). There are global standards on privacy and security management (ISO/IEC 27701, ISO 27799), privacy impact assessment (ISO/IEC 29134), pseudonymization and de-identification techniques (ISO 25237, ISO/IEC 20889), on secure health software development lifecycle (ISO/IEC 62304), or other standards such as data protection by design (prEN 17529) or more recent standards on the International Patient Summary and its implementation in Europe (EN ISO 27269 and CEN/TS 17288).

### Controversies on Data Sharing Outside Europe: Schrems Cases

Although GDPR governs the data transfer between the EEA and external countries, significant doubt has arisen concerning the legitimacy of transferring personal data to countries outside the EEA area. One of the most known cases is the Schrems II case which highlighted some of these challenges and led to the invalidation of the Privacy Shield as a mechanism for transferring data from Europe to the United States on July 16, 2020.^12^ The Privacy Shield was a self-sign certification in which US companies certify to the US Department of Commerce that they meet the data protection standards (eg, GDPR). In response to the court case, the European Commission has proposed the Standard Contractual Clauses to regulate data transfer from the EU/EEA (subject to the GDPR) to entities outside the EU/EEA that are not subject to the GDPR.

The information about data transfer in Europe must be available to the users (eg, patients). This information is often available via the Terms of Service (ToS) and Privacy Policy documents made by the processor of the data (eg, manufacturer).

## Objective

This study aims to analyze the mandatory ToS and Privacy Policy documents for medical equipment used by individuals with diabetes, to existing regulations regarding data sharing. To guide our analysis, we formulated 2 research questions:

**Research Question 1:** How do ToS and Privacy Policy regulate the data flow from the patients’ medical equipment to the manufacturers, third parties, and countries outside EEA?
**Research Question 2:** How do HCPs access patient-gathered data?

## Materials and Methods

We performed a Document Analysis^13^ to summarize findings from the ToS and Privacy Policy documents.

### Documents Sources and Search Strategy

We only considered the medical equipment devices available for individuals with diabetes in Norway that are listed in the purchasing agreement between the Norwegian Regional Health Authorities and the vendors from October 1, 2022 to September 30, 2023.^14^ Based on the medical devices listed, we performed multiple data searches in October 2022 for the documents referencing the ToS and Privacy Policy. Then, we approached each medical supplier listed in the National Agreement for confirmation about the document identified.

### Identification and Evaluation Key Elements

We investigated the documents provided by vendors/manufacturers (after searching contact via e-mails and phone calls) or those to which we were referred to online. Regrettably, some medical suppliers listed in the national agreement did not respond to our inquiries, and for those, we used the ones identified by online search. Afterwards, we identified and evaluated related software that regulates the data flow from all the eligible medical devices.

**Figure 2.**
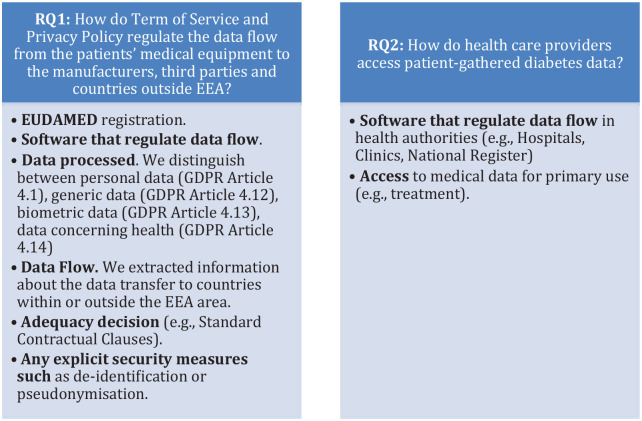
Document analysis key elements. Abbreviations: EEA, European Economic Area; EUDAMED, European Database on Medical Devices; GDPR, General Data Protection Regulation.

The authors (MP and DT) have extracted multiple items for the identified ToS and Privacy Policy documents. All the authors agreed upon the analysis of the elements reported in Figure 2 in line with the analysis objectives.

## Results

### Medical Equipment Identified

We identified 11 different medical equipment distributed by Norwegian Regional Health Authorities,^14^ reported in Table 1.

**Table 1.** Insulin Pumps, CGMs, and Hybrid Closed-Loop Systems Available for Patients in Norway.

Medical equipment (n = 11)	Categories
MiniMed 780G + Guardian Connect G4	Insulin pump with hybrid closed-loop technology
MiniMed 640G^a^ + Guardian Connect G3	Insulin pump with Predictive Low-Glucose Suspend (PLGS)
Tandem t:slim X2 Insulin pump Control-IQ technology + Dexcom G6	Insulin pump with hybrid closed-loop technology
OmniPod Dash	Insulin patch pump
Accu-Chek Solo	Insulin patch pump
Accu-Chek Insight^a^	Insulin pump
Guardian Connect G4	Stand-alone CGM
FreeStyle Libre 2	Stand-alone CGM
FreeStyle Libre 3	Stand-alone CGM
Dexcom G6	Stand-alone CGM
Eversense E3	Stand-alone CGM

Abbreviation: CGM, continuous glucose monitoring.

a Supported until April 2023.

#### Medical equipment registration in the European Database on Medical Devices database

Only 3 of the 11 diabetes devices studied have been registered in the EUDAMED database. The OmniPod Dash has been classified as a Class IIb risk under the MDR. In addition, both the Accu-Chek Insight and Accu-Chek Solo have been registered under Annex II List B of the IVDR.

### Data Flow From Medical Equipment to Patients and Health Care Providers

Vendors of several medical devices require patients to use their smartphones to display measured health information. Patients who lack access to a smartphone or choose not to use one are referred to built-in monitoring systems, such as the FreeStyle Libre 2 and Dexcom G6 which have a dedicated data reading device.^14^

Table 2, which supplements Table 1, illustrates potential software additions for the identified medical equipments in Europe. Notably, several of these software applications may be compatible with multiple devices, whereas the Privacy Policies and ToS documents may have joint applicability to more than one software application.

**Table 2.** Software Applications for the Medical Equipment.

Medical equipment (n = 11)	Software that regulate the data flow (users) (n = 12)	References
MiniMed 780G	CareLink Connect (HCP), MiniMed Mobile (P), Guardian Connect (P)	^15-18^
MiniMed 640G		
Guardian Connect G4		
Tandem t:slim X2 Insulin pump Control-IQ technology	t:connect mobile (P, HCP), Glooko (P, HCP)	^19-22^
OmniPod Dash	Omnipod Display (P), Glooko (P, HCP)	^21-24^
Accu-Chek Solo	mySugr (P), RocheDiabetes Care Platform (HCP), Glooko (P, HCP)	^21,22,25-28^
Accu-Chek Insight		
FreeStyle Libre 2	LibreView Data Management System (HCP), FreeStyle App (P)	^29-32^
FreeStyle Libre 3		
Dexcom G6	Dexcom Clarity (P, HCP), Glooko (P, HCP)	^21,22,33,34^
Eversense E3	Contour Diabetes (P)	^35,36^

Abbreviations: P, patient; HCP, health care provider.

#### Software registration for health care providers and European Database on Medical Devices database

At present, patient-gathered data from medical equipment and apps cannot be directly downloaded into the electronic health record (EHR) systems used in Norwegian hospital clinics. As a result, HCPs need to access patient data through other software. In Table 2, we analyzed the software that can be used to access patient data and identified 6 options for the clinics: CareLink Connect, LibreView Data Management System, RocheDiabetes Care Platform, t:connect mobile, Dexcom Clarity, and the only data aggregator Glooko is compatible with multiple devices. Furthermore, when examining the related software, we found that none (0/12) of these software applications are registered as medical devices in the EUDAMED database.

### Overview of Data Processed by Software

The software that regulate the data flow (n = 12), previously identified in Table 2, collect and process different data. In Table 3, we present an overview of the software processing health-related data (GDPR Article 4.14). All software applications collect personal data (GDPR Article 4.1), whereas only Glooko^12,13^ and t: connect mobile^8,9^ collect biometric data.

**Table 3.** Data Processed From Various Software.

Software that regulate data flow (users)	Data concerning health	Specific security measures	Adequacy decisions^a^	Reference to documents (ToS, Privacy Policy)
Glooko (P, HCP)	X	GDPR-compliant anonymization, encryption	Standard contractual clauses, privacy shield, Binding Corporate Rules (BCRs)	^21,22^
MiniMed Mobile (P) Guardian Connect (P)	X	GDPR compliance	Standard contractual clauses	^16,18^
CareLink Connect (HCP)		GDPR compliance, possibly pseudonymization, anonymization, and encryption	Adequacy decision or else, standard contractual clauses	^15,17^
t:connect (P, HCP)	X	GDPR compliance, encryption, access control, event logging	None	^19,20^
OmniPod DISPLAY (P) OmniPod VIEW (HCP)	X	GDPR compliance	None	^23,24^
mySugr (P)	X	Specific security measures such as Data transfer via HTTPS (hypertext transfer protocol secure), user can operate via pseudonym, and anonymization	Standard contractual clauses	^25,26^
RocheDiabetes Care Platform (HCP)		GDPR compliance, access control	Standard contractual clauses	^27,28^
LibreView Data Management System (HCP)		GDPR compliance, access control, de-identifying, pseudonymizing, aggregating, and/or anonymizing the personal information	Compliance with laws of patient’s jurisdiction	^30,31^
FreeStyle App (P)	X	De-identify, pseudonymize, aggregate and/or anonymize, encrypted Bluetooth connections for FreeStyle Libre sensors, 2-factor authentication for LibreView users	None	^29,32^
Dexcom Clarity (P, HCP)	X	GDPR compliance, transmission encrypted	Standard contractual clauses	^33,34^
Contour Diabetes (P)	X	GDPR compliance, encryption, anonymized, or de-identified/pseudonymized information	Standard contractual clauses	^35,36^

Abbreviations: ToS, Terms of Service; P, patient; HCP, health care provider; GDPR, General Data Protection Regulation.

a An “adequacy decision” is a decision made by the European Commission (EU) that recognizes that a non-EU country or organization provides the same level of protection for personal data as the EU does.

As follows, we provide an overview of the specific security measures identified. All software applications use third-party service providers to deliver their services, such as information technology and hosting services. Table 3 also presents the legal basis for data export to non-European jurisdictions under “Adequacy decisions.”

## Discussion

### Main Findings

We identified 11 types of medical equipment used by diabetes patients in Norway (Table 1). To analyze how HCPs access patient diabetes data (RQ2), we identified software that regulates data flow (n = 12) (Table 2). Some software applications can be used by both patients and HCPs (3/12), whereas others are used exclusively by 1 group (6/12 by patients, 3/12 by HCPs).

We analyzed compliance with GDPR security measures (RQ1) and found that some software relies on adequacy decisions (8/12). The remaining 4 software applications did not specify any adequacy decisions (4/12).

We also investigated the registration status of medical equipment and software in the EUDAMED database to comply with the new MDR and IVDR regulations. Only 3 devices (OmniPod Dash, Accu-Chek Insight, and Accu-Chek Solo) were registered in EUDAMED, but none of their respective software applications (RQ1).

### Perceived Necessity vs Policy Overload: A Dilemma for Medical Equipment Users

While a smartphone is not strictly necessary for managing diabetes, it can be helpful due to the ability of mobile apps and software to facilitate glucose monitoring and automatic data recording and data transfer. Medical equipment used for diabetes management includes Bluetooth or Near-Field Communication (NFC) tags for wireless communication with smartphones.^37^ Alternative devices can be provided for patients who choose not to use a mobile phone.

Patients who use vendor software applications are required to acknowledge and accept the ToS and Privacy policies.^15-36^ In addition, patients must provide informed consent for the processing of their data.^38^ However, the documents governing the use of these software applications can often be intricate and broad, presenting, creating a dilemma for users who may simply decide that the benefits outweigh the challenges of navigating these lengthy documents.

Future studies should investigate the different sensitivity of users toward data sharing, the perceived need for this technology, and the impact on the acceptance of these terms.

### Data-Sharing Challenges for Primary and Secondary Use of Data

The medical equipment outlined in Table 1 play a crucial role in health care, and many software applications are widely used for planning the treatment of patient (primary use of data). However, none of these applications are directly integrated into the EHR system, which creates a challenge for HCPs who must use multiple systems with different login processes and platforms. This can take up valuable time during consultations, potentially affecting the quality of patient care.^5,39-41^ Furthermore, it is important to note that these systems, in their current state, are not designed for integration with EHR. The systems do not intend to be an EHR, as exemplified by the LibreView data management system’s declaration: “THE LIBREVIEW DATA MANAGEMENT SYSTEM IS NOT AN ELECTRONIC HEALTH RECORDS SYSTEM AND YOU MUST PRINT AND/OR DOWNLOAD PATIENT INFORMATION THAT YOU DEEM RELEVANT TO YOUR PROVISION OF MEDICAL CARE, TREATMENT OR ADVICE.”^30,31^ The manual process of transferring data from the data management systems into EHRs can increase the risk of errors and create inefficiencies in the data reporting process.^6^

When it comes to sharing data for secondary use, the GDPR grants patients the right to receive personal data in a machine-readable format (ART.20 Rights to data portability). However, patients and informal caregivers often face difficulties when attempting to download diabetes data.^42,43^ These challenges bring into question the ownership of patient data, as it remains largely within the medical vendor ecosystem.

Thus, the diverse data structures used by medical equipment manufacturers make integrating or sharing data directly into EHR systems or for research studies challenging. To mitigate these issues, the adoption of a common data exchange standard like Fast Healthcare Interoperability Resources (FHIR) is essential.

#### The controversy about whether software applications should be considered as medical device

None of the software applications listed in Table 2 is registered as medical devices in the EUDAMED database. We have identified 2 different potential reasons. The first one could be due to the disclaimers presented to patients, such as “No medical advice: THE LIBREVIEW DATA MANAGEMENT SYSTEM IS NOT INTENDED FOR THE DIAGNOSIS OF OR SCREENING FOR DIABETES MELLITUS”^30^ or “YOUR USE OF THE SERVICE IS SOLELY AT YOUR OWN RISK.”^21^

While disclaimers might reduce the legal obligations of software providers, it is crucial to prioritize their intended use. Moreover, disparities in software registration as medical devices could give rise to issues. The absence of medical device registration might spark controversy, especially when these software applications are used or endorsed within hospital premises and can be perceived as medical devices.

Ultimately, the effectiveness of EUDAMED will need to be evaluated once it is fully implemented as it will become mandatory in 2026.^10^ This database includes a module for reporting severe events related to devices and corrective safety measures. Besides the intended use of the software, including digital health applications in this module is challenging due to the constantly evolving nature of Information and Communications Technology (ICT) data security and managing multiple security risks.^44^

### Technical Overview and How Data Are Shared

Although the legal documents provide details about the data processed by the software, they often lack specific and detailed security measures. The documents primarily offer recommendations for password handling and highlight the responsibility of professional users to protect their accounts.^30,31^

Data sharing between software applications can complicate the understanding of how patient health information is processed. Patient software applications may collect and process health information, which is then accessed by HCPs software through a cloud solution without further processing. We could assume that as the software exclusive for HCPs, as indicated in Table 3, do not collect any health data. Furthermore, there is a lack of comprehensive information regarding the specific categories of data processed, the manner in which data flows, how long it is stored, techniques employed for de-identification, encryption protocols, and data formats.

Finally, it is important to understand the ToS and Privacy Policies for any third-party applications before opting in and consenting to sharing data with them. For example, once data are shared with a third-party application, the provider, or the patient, no longer controls its use, access, or disclosure.^21,22^ Abbott, for instance, uses cloud providers like Amazon Web Services and Microsoft Azure.

### Limitations

The presented analysis has some limitations, such as restricting the devices to those available in Norway and that we did not receive adequate feedback from all the vendors. Nevertheless, the work is still relevant for the entire EEA/EU area because Norway is part of the EEA Board without a voting right for GDPR-related matters. General Data Protection Regulation and the security and privacy issues discussed are also highly relevant for those outside EEA/EU. It is important to note that the list of compatible apps (described in Table 2) may evolve over time, and this study only examines those available during the specified period.

## Conclusions and Implications for the Future

The current state of medical device technology development is largely dominated by companies outside of the European Economic Area (EEA).

This study is the first to analyze the ToS and Privacy Policy documents for diabetes medical equipment that national authorities have approved. These documents are not easy to understand to end-users and require a high level of legal and digital literacy, as indicated by a previous study.^45^ Due to complex or legalistic terminology, most users may consent without adequately understanding the terms and conditions presented online.^46,47^ Future research should explore users’ levels of sensitivity toward data sharing, their perceived necessity for this technology, and their acceptance of the related terms and conditions.

Future research should also investigate how to effectively educate and train health care professionals on data security and privacy to increase their awareness and understanding of these issues,^48^ as HCPs prioritize functionalities over security and privacy concerns when recommending these tools to patients.^49^ A standardized health care data-sharing approach (eg, FHIR) could integrate these tools into existing EHR systems. This would simplify the work of health care providers in their clinical practice as they would no longer need to interact with multiple systems and procedures to access and view patient data.
